# The immune receptor FcRγ-chain mediates CD36-induced platelet activation and thrombosis by oxidized low-density lipoproteins^∗^

**DOI:** 10.1182/bloodadvances.2024015652

**Published:** 2025-07-18

**Authors:** Katie S. Wraith, Jawad S. Khalil, Ahmed A. Aburima, Lih T. Cheah, Matthew S. Hindle, Martin Berger, Romez Uddin, Hoor Ayub, Mary McKay, Rui-Gang Xu, Robert A. S. Ariëns, Mark T. Kearney, Michael G. Tomlinson, Khalid M. Naseem

**Affiliations:** 1 Biomedical Institute for Multimorbidity, Centre for Biomedicine, Hull York Medical School, University of Hull, Hull, United Kingdom; 2 Leeds Institute of Cardiovascular and Metabolic Medicine, University of Leeds, Leeds, United Kingdom; 3 Department of Internal Medicine, University Hospital Aachen, Aachen, Germany; 4 School of Biosciences, University of Birmingham, Birmingham, United Kingdom; 5 Centre of Membrane Proteins and Receptors, Universities of Birmingham and Nottingham, Birmingham, United Kingdom

## Abstract

The scavenger receptor CD36 links atherogenic dyslipidemia to platelet hyperactivity and accelerated thrombosis through the binding of oxidized low-density lipoproteins (oxLDL). The signaling mechanism(s) that facilitates CD36 translation of oxidative lipid stress into platelet activation is unclear. We examined the role of immunoreceptor tyrosine-based activation motif (ITAM) adapter proteins in CD36-mediated platelet activity. oxLDL induced the phosphorylation of the ITAM-containing adapter Fc receptor γ-chain (FcRγ) in human and murine platelets. Phosphorylation of FcRγ was blocked by Src family kinase (SFK) inhibitors, mimicked by CD36-specific oxidized phospholipids and ablated in CD36^−/−^ murine platelets. Under basal conditions, a pool of CD36 formed a multiprotein complex that included FcRγ and the SFKs Lyn and Fyn. CD36 ligation by oxLDL resulted in the recruitment, phosphorylation, and activation of the tyrosine kinase Syk. To explore the functional cooperativity of this CD36-FcRγ complex, we used murine platelets deficient in FcRγ. The genetic ablation of FcRγ prevented oxLDL-induced tyrosine phosphorylation of Syk and downstream adapter SLP-76, but not SFKs. Moreover, platelet aggregation, in vitro thrombosis, and in vivo carotid thrombosis stimulated by oxLDL were lost in the absence of FcRγ. This study establishes FcRγ as a first functional coreceptor for CD36 in platelets, which enables lipid platelet hyperactivity and arterial thrombosis.

## Key Points

• A pool of platelet CD36 is constitutively associated with FcRγ.
• Ligation of CD36 by oxLDLs phosphorylates FcRγ and recruits tyrosine kinase Syk, leading to platelet hyperactivity and thrombosis in mice.

## Visual Abstract

**Figure:**
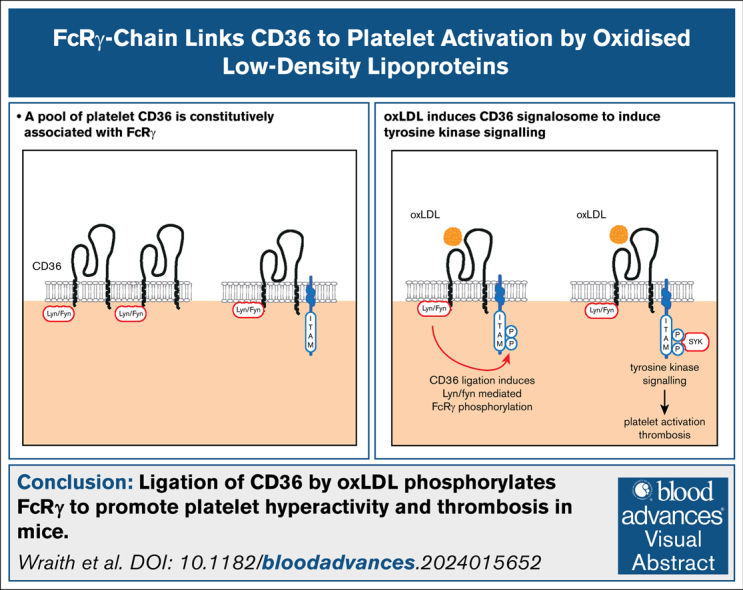

## Introduction

Dyslipidemia in humans is associated with heightened platelet activity, characterized by increased levels of platelet-monocyte complexes, elevated P-selectin expression on platelets, and an enhanced risk of thrombosis.^1^^,^^2^ Experimental dyslipidemia in mice similarly gives rise to a prothrombotic phenotype.^3^ In both human and murine studies, oxidized low-density lipoprotein (oxLDL) particles are recognized as potential contributors to platelet hyperactivity in dyslipidemia. oxLDL have been detected in the plasma of individuals with dyslipidemia, acute coronary syndromes, ischemic stroke, and obesity, and are associated with adverse outcomes.^4^^,^^5^ Studies have consistently shown that these particles exhibit the capability to augment multiple platelet-activating functions in both in vitro and ex vivo settings.^6^, ^7^, ^8^ Consequently, unraveling the molecular mechanistic underpinnings of the interaction between these circulating atherogenic particles and platelets may yield valuable insights into the mechanisms driving platelet hyperactivity in atherothrombotic diseases.

The complex structure of oxLDL generates particles with multivalent properties that interact with several platelet receptors, including CD36, scavenger receptor A, platelet-activating factor receptor, and lectin-like oxLDL receptor 1.^9^, ^10^, ^11^, ^12^ Among these receptors, CD36 has emerged as a prominent candidate for oxidative lipid stress–induced platelet hyperactivity through its binding to oxLDL, very low-density lipoprotein, microparticles, advanced glycation end products, S100-A9, proprotein convertase subtilisin/kexin type 9, and the COVID-19 spike protein.^10^^,^^13^, ^14^, ^15^, ^16^ We, and others, have shown that CD36-mediated platelet activation involves tyrosine kinase signaling pathways.^7^^,^^9^ However, deciphering the precise signaling mechanisms of CD36 is challenging because of its promiscuous ligand binding and the absence of conventional signaling motifs within the intracellular domains of CD36. In many cell types, CD36 facilitates ligand-dependent responses by forming diverse heteromeric signaling complexes including functional interactions with Toll-like receptor 2 (TLR2), TLR4, TLR6, CD47, β1 integrin, and Fc receptor γ-chain (FcRγ).^17^ However, in platelets, CD36 has only been shown to associate with CD9, which also lacks signaling motifs.^18^ Given the absence of traditional CD36-associated signaling motifs, the mechanisms enabling signaling after ligation of CD36 have remained unclear.

The canonical platelet-activating tyrosine kinase cascade typically begins with the phosphorylation of an immunoreceptor tyrosine-based activation motif (ITAM)–containing adapter by Src family tyrosine kinases (SFKs). Human platelets express 2 ITAM-containing proteins, the FcRγ and FcγRIIA (required for glycoprotein VI (GPVI) and α_IIb_β_3_ signaling, respectively), and the hemi-ITAM (hemITAM)-containing C-type lectin-like receptor 2.^19^, ^20^, ^21^ These ITAMs recruit the nonreceptor tyrosine kinase spleen tyrosine kinase (Syk) via their tandem phosphorylated YXXL motifs. The activation of Syk leads to phospholipase Cγ2 (PLCγ2) activation, calcium mobilization, and integrin activation.^19^ Considering the established association between CD36 and tyrosine kinase signaling in platelets, we hypothesized that ITAM signaling could contribute to the translation of plasma lipid stress into platelet hyperactivity. Here, we reveal the presence of a novel platelet CD36 interactome that includes the SFKs Lyn and Fyn, and FcRγ. Engagement of CD36 by oxLDL induces the phosphorylation of this ITAM in an SFK-dependent manner, leading to Syk recruitment and phosphorylation. Genetic deletion of FcRγ prevents oxLDL-induced tyrosine kinase signaling and platelet activation, and diminishes thrombosis both in vitro and in vivo.

## Materials and methods

### Materials

PP2 and PP3 were from Calbiochem (Nottingham, United Kingdom). Dasatinib was from Selleckchem (Suffolk, United Kingdom). KOdiA-PC (1-(palmitoyl)-2-(5-keto-6-octene-dioyl) phosphatidylcholine; subsequently referred to as oxPC_CD36_) and PAPC (1-palmitoyl-2-arachidonoyl-sn-glycero-3-phosphocholine), and antibodies to Syk (4D10), Lyn, CD36 (H-300), and CD36 (N-15) were from Santa Cruz (Wembley, United Kingdom). Wheat germ agglutinin, Alexa Fluor 488 was from Fisher Scientific (Loughborough, United Kingdom). Collagen-related peptide was from the University of Cambridge (Cambridge, United Kingdom). Duolink In Situ kit was from Sigma (Poole, United Kingdom). Antibodies to FcRγ, phospho-tyrosine (phospho-tyrosine; 4G10), β-tubulin, and mouse immunoglobulin G isotype control were from Upstate (Watford, United Kingdom). Antibodies to Fyn, phospho–Src-tyr^416^, phospho–Syk-tyr^352^, and rabbit immunoglobulin G isotype control were from Cell Signaling Technology Inc (Hitchen, United Kingdom). Antibodies to phospho–SLP-76-tyr^128^, protein kinase A (PKA) regulatory subunit II (RII), fluorescein isothiocyanate (FITC)–P-selectin, FITC-CD49b, and FITC-CD61 were from BD Biosciences (Oxford, United Kingdom). Antibodies to FITC-CD42b and FITC-GPVI were from Emfret (Eibelstadt, Germany). Antibodies to Syk (5F5) and phycoerythrin-CD36 were from BioLegend (Cambridge, United Kingdom). CD36 (FA6.152) antibody was from STEMCELL Technologies (Cambridge, United Kingdom). Convulxin was from Cayman Chemical (United Kingdom). Anti-FcγRIIA antibody was kindly provided by Jim Robinson (University of Leeds).

### Experimental animals

FcγRIIA^+/+^, GPVI^−/−^ (provided by Steve Watson, University of Birmingham, Birmingham, United Kingdom), CD36^−/−^, and wild-type (WT; Charles River, Kent, United Kingdom) animals were all on a C57BL/6 background. All mice (both male and female were used) were fed a normal chow diet and used for experimentation at 12 weeks of age as approved by the United Kingdom Home Office.

### LDL isolation and oxidation

LDL (density, 1.019-1.063 g/mL) was prepared from fresh human plasma by sequential density ultracentrifugation and oxidized with CuSO_4_ (10 μmol/L),^7^ with separate LDL preparations used to repeat the individual experiments. Oxidation was determined by measurement of relative electrophoretic mobility on agarose gels. Relative electrophoretic mobility was calculated for nonoxidized control native LDL (nLDL) (1) and oxLDL (3.58 ± 0.23; *P* < .05 compared with nLDL).

### Platelet aggregation, flow assays, flow cytometric analysis, intravital microscopy, NFAT assay, immunoprecipitation, immunoblotting, and proximity ligation assay (PLA)

Detailed methods are presented in the supplemental Methods.

### Statistical analysis

Experimental data were analyzed by GraphPad Prism 6 (La Jolla, CA). Data are presented as mean ± standard error of the mean of at least 3 different experiments. Differences between groups were calculated using Mann-Whitney *U* test or Kruskal-Wallis test for nonparametric testing and statistical significance accepted at *P* ≤ .05.

All studies were approved by the Hull York Medical School ethics committee and the University of Leeds research ethics committee. Blood donors provided informed consent in accordance with the Declaration of Helsinki.

## Results

### OxLDL induces CD36-dependent sequential phosphorylation of SFK, FcRγ, and Syk

**Figure 1.**
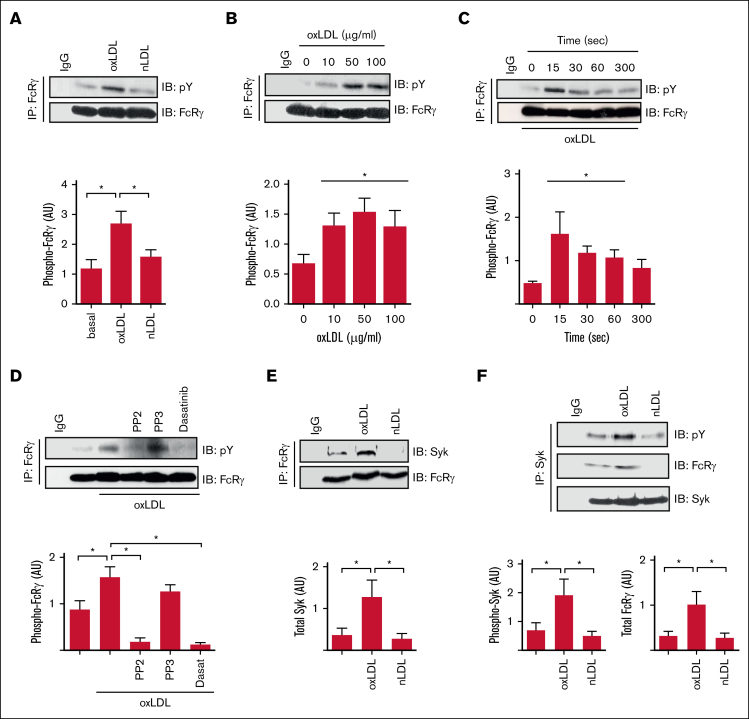
**oxLDL stimulates tyrosine phosphorylation of the FcRγ.** (A) Washed human platelets (7 × 10^8^/mL) were either untreated or treated with oxLDL (50 μg/mL) or nLDL (50 μg/mL) for 15 seconds. FcRγ was immunoprecipitated from lysates and immunoblotted for phospho-tyrosine and FcRγ; representative blots (upper) and densitometric analysis (lower) of 5 independent experiments. ∗*P* < .05. (B) As in panel A, except platelets were stimulated with increasing concentrations of oxLDL (10-100 μg/mL). Representative blots (upper) and densitometric analysis (lower) of 5 independent experiments. ∗*P* < .05. (C) As in panel A, except platelets were stimulated with oxLDL (50 μg/mL) for up to 300 seconds. Representative blots (upper) and densitometric analysis (lower) of 3 independent experiments. ∗*P* < .05. (D) As in panel A, except platelets were treated with either PP2 (20 μM), PP3 (20 μM), or dasatinib (10 μM) for 3 minutes before stimulation with oxLDL (50 μg/L; 15 seconds). Representative blots (upper) and densitometric analysis (lower) of 4 independent experiments. ∗*P* < .05. (E) As in panel A, except immunoblotted for Syk and FcRγ. Representative blots (upper) and densitometric analysis (lower) of 10 independent experiments. ∗*P* < .05. (F) As in panel A, except Syk was immunoprecipitated and immunoblotted for phospho-tyrosine, FcRγ, and Syk. Representative blots (upper) and densitometric analysis for phospho-Syk (lower-left) and FcRγ (lower-right) of 6 independent experiments. ∗*P* < .05. AU, arbitrary units; IB, immunoblot; IgG, immunoglobulin G; IP, immunoprecipitate.

CD36-mediated activation of platelets requires SFKs, Syk, and PLCγ2,^7^^,^^9^ resembling GPVI and integrin α_IIb_β_3_, which require an ITAM-containing protein to link SFKs to Syk.^19^ Given that CD36 does not possess known signaling motifs, we hypothesized that this thrombotic receptor signaled through an ITAM-linked pathway. Human platelets express 2 major ITAM-containing proteins, FcRγ and FcγRIIA, that could potentially facilitate CD36 signaling. Because FcRγ can associate with CD36 in macrophages,^22^ we focused on this as a potential adapter. Immunoprecipitation of FcRγ from human platelet lysates revealed that it was tyrosine phosphorylated in response to oxLDL but not the nonoxidized control nLDL (Figure 1A). OxLDL was effective at a range of concentrations (10-100 μg/mL) but maximal at 50 μg/mL (Figure 1B). FcRγ phosphorylation was rapid, occurring within 15 seconds of exposure to oxLDL (50 μg/mL) and maintained for up to 60 seconds before returning to basal level (Figure 1C). Treatment of platelets with 2 distinct SFK inhibitors, PP2 (20 μM) and dasatinib (10 μM), but not the inactive analog PP3, abolished phosphorylation of FcRγ (Figure 1D), indicating that it lies downstream of SFK. The tyrosine phosphorylation of FcRγ facilitates the recruitment and phosphorylation of the tyrosine kinase Syk in platelets in response to collagen.^19^ Treatment of platelets with oxLDL (50 μg/mL), but not nLDL, increased the association of FcRγ with Syk (Figure 1E). Reverse immunoprecipitation confirmed oxLDL induced both the phosphorylation of Syk and its interactions with FcRγ (Figure 1F), suggesting that FcRγ may play a role in the early signaling events required for tyrosine kinase mediated platelet activation in response to oxLDL.

### OxLDL induces phosphorylation of FcRγ, but not FcγRIIA, in a CD36 dependent manner

**Figure 2.**
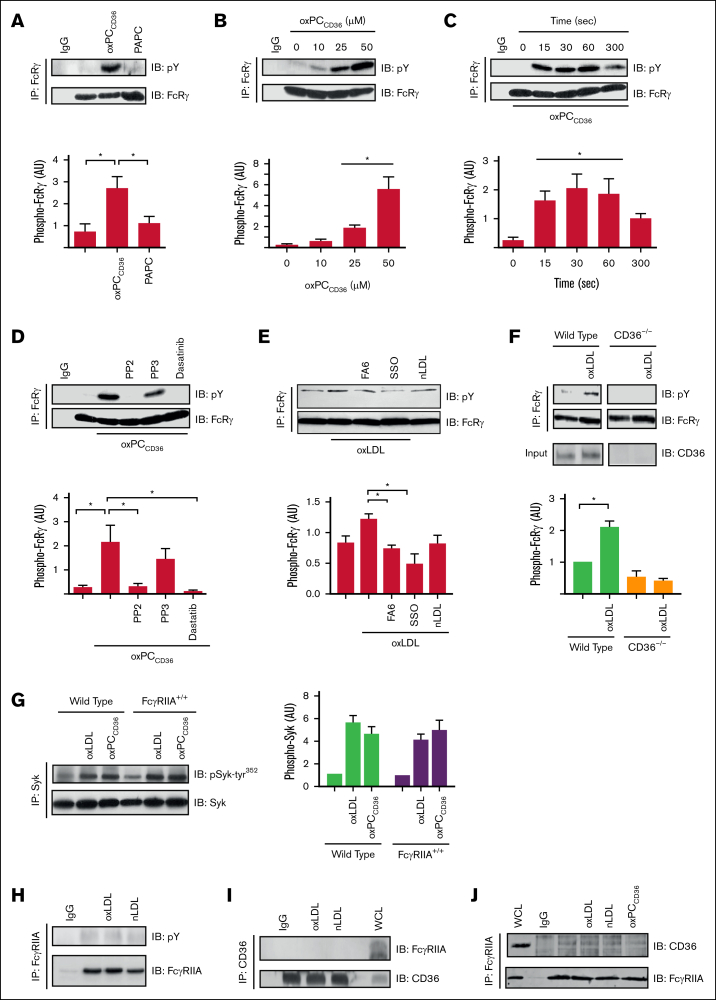
**CD36 stimulation results in tyrosine phosphorylation of the FcRγ.** (A) Washed human platelets (7 × 10^8^/mL) were either untreated or treated with oxPC_CD36_ (25 μM) or PAPC (25 μM) for 15 seconds. FcRγ was immunoprecipitated and immunoblotted for phospho-tyrosine and FcRγ. Representative blots (upper) and densitometric analysis (lower) of 6 independent experiments. ∗*P* < .05. (B) As in panel A, except platelets were stimulated with increasing concentrations of oxPC_CD36_ (10-50 μM) for 15 seconds. Representative blots (upper) and densitometric analysis (lower) of 3 independent experiments. ∗*P* < .05. (C) As in panel A, except platelets were stimulated with oxPC_CD36_ (25 μM) for up to 300 seconds. Representative blots (upper) and densitometric analysis (lower). ∗*P* < .05. (D) As in panel A, except platelets were pretreated with either PP2 (20 μM), PP3 (20 μM), or dasatinib (10 μM) for 3 minutes, followed by stimulation with oxPC_CD36_ (25 μM; 15 seconds). Representative blots (upper) and densitometric analysis (lower) of 4 independent experiments. ∗*P* < .05. (E) As in panel A, except platelets were pretreated with FA6-152 (1 μg/mL) or SSO (50 μM) for 5 minutes, followed by stimulation with oxLDL (50 μg/mL; 15 seconds). Representative blots (upper) and densitometric analysis (lower) of 3 independent experiments. ∗*P* < .05. (F) Washed platelets (7 × 10^8^/mL) from WT and CD36^−/−^ mice, either untreated or treated with oxLDL (50 μg/mL) for 30 seconds, were lysed; FcRγ was immunoprecipitated and immunoblotted for phospho-tyrosine, FcRγ, and CD36. Representative blots (upper) and densitometric analysis (lower) of 3 independent experiments. ∗*P* < .05. (G) Washed platelets (5 × 10^8^/mL) from either WT or FcγRIIA^+/+^ mice were untreated or treated with oxLDL (50 μg/mL) or oxPC_CD36_ (50 μM) for 1 minute. Platelets were lysed; Syk was immunoprecipitated and immunoblotted for phospho–Syk-tyr^352^ and Syk. Representative blots (left) and densitometric analysis of phospho-Syk (right) of 3 independent experiments. ∗*P* < .05. (H) Washed human platelets (5 × 10^8^/mL) were untreated or treated with oxLDL (50 μg/mL) or nLDL (50 μg/mL) for 1 minute. Platelets were lysed; FcγRIIA was immunoprecipitated and immunoblotted for phospho-tyrosine and FcγRIIA. Representative blots of 3 independent experiments. (I) As in panel H, except platelets were lysed; CD36 was immunoprecipitated and immunoblotted for FcγRIIA and CD36. Representative blots of 3 independent experiments. (J) As in panel H, except platelets were also stimulated with oxPC_CD36_ (25 μM), and lysed, and FcγRIIA was immunoprecipitated and immunoblotted for CD36 and FcγRIIA. Representative blots of 3 independent experiments. AU, arbitrary units; IgG, immunoglobulin G; pY, phospho-tyrosine; SSO, sulfosuccinimidyl oleate.

To establish whether CD36 facilitated the phosphorylation of FcRγ in response to oxLDL, we used a 3-pronged approach. First, we found that oxPC_CD36_, a CD36-specific oxidized phospholipid present in oxLDL^23^ but not the nonoxidized control phospholipid PAPC, induced tyrosine phosphorylation of FcRγ in a concentration-dependent manner (Figure 2A-B) with similar kinetics of phosphorylation induced by the parent oxLDL particles (Figure 1., Figure 2.C). The phosphorylation of the FcRγ by oxPC_CD36_ was SFK dependent because it was blocked by PP2 and dasatinib (Figure 2D). Second, oxLDL failed to increase the phosphorylation of FcRγ in platelets treated with the CD36 blockers FA6-152 (1 μg/mL) and sulfosuccinimidyl oleate (50 μM; Figure 2E). Third, oxLDL induced the phosphorylation of FcRγ in WT murine platelets but not in platelets deficient in CD36 (Figure 2F; supplemental Figure 1).

FcγRIIA is a second ITAM present in human, but not in murine platelets, in which it facilitates the phosphorylation and activation of Syk in response to ligation of integrin α_IIb_β_3_.^20^ We explored the possibility that FcγRIIA contributed to platelet tyrosine kinase signaling in response to oxLDL. OxLDL and oxPC_CD36_ induced phosphorylation of Syk (phospho–Syk-tyr^352^) in WT mice (absence of FcγRIIA), which was unchanged in platelets expressing FcγRIIA as a transgene^24^ (Figure 2G), confirming the potential for Syk activation in the absence of FcγRIIA. In addition, treatment of human platelets with oxLDL did not lead to the phosphorylation of FcγRIIA (Figure 2H). Furthermore, immunoprecipitation of CD36 from human platelets did not result in the coprecipitation of FcγRIIA, which was confirmed through reverse immunoprecipitation experiments (Figure 2I-J).

### OxLDL induce the formation of a CD36-SFK-ITAM signalosome

**Figure 3.**
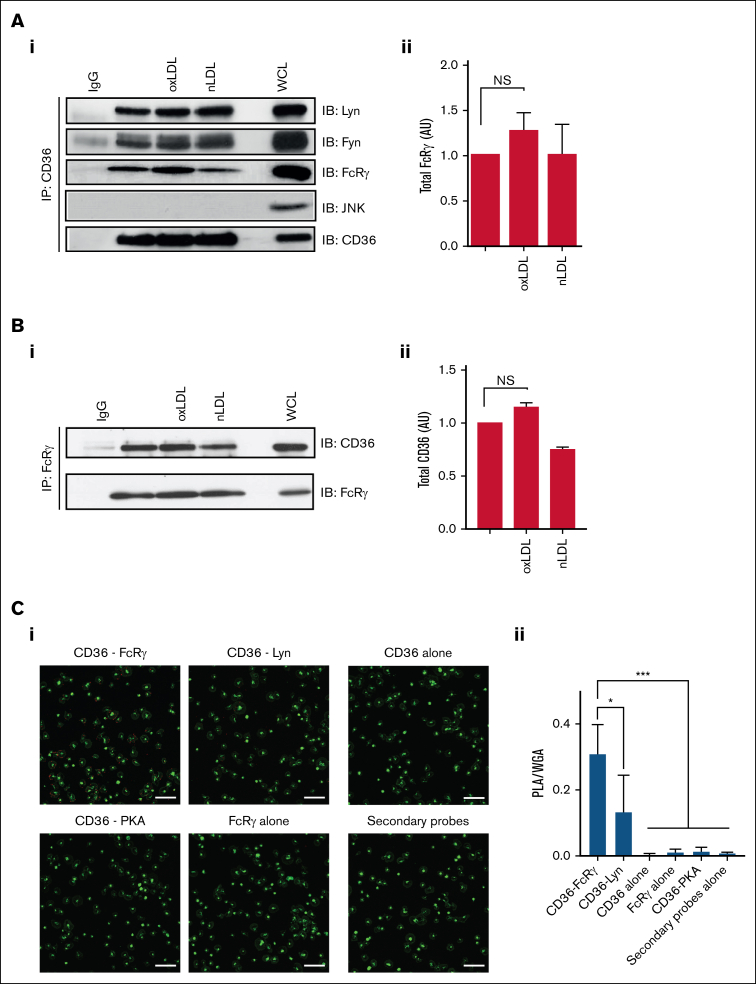
**CD36 is associated with the FcRγ in human platelets.** (A) Washed human platelets (7 × 10^8^/mL), either untreated or treated with oxLDL (50 μg/mL) or nLDL (50 μg/mL) for 15 seconds, were immunoprecipitated for CD36 and immunoblotted for Fyn, Lyn, FcRγ, JNK, and CD36. (Ai) Representative blots (i) and densitometric analysis of FcRγ from 7 independent experiments (ii). (B) Washed human platelets (7 × 10^8^/mL), either untreated or treated with oxLDL (50 μg/mL) or nLDL (50 μg/mL) for 15 seconds, were immunoprecipitated for FcRγ and immunoblotted for CD36. (i) Representative blots and (ii) densitometric analysis of CD36 from 4 independent experiments. (C) Washed human platelets (3 × 10^6^/mL) were adhered to glass coverslips coated with oxLDL (100 μg/mL) and incubated with combinations of CD36 and FcRγ antibodies; CD36 and Lyn antibodies; CD36 and PKA RII antibodies; CD36 antibody alone; FcRγ antibody alone; or antibody diluent alone. Platelets were then stained with Alexa Fluor 488–conjugated wheat germ agglutinin to identify membranes (green) and PLA probes to detect protein-protein interactions (red). Samples were viewed using confocal microscopy under ×63 original magnification. Scale bar, 20 μm. (i) Representative overlaid images and (ii) quantification of PLA/WGA from 4 independent experiments. ∗*P* < .05, ∗∗∗*P* < .001. IgG, immunoglobulin G; NS, nonsignificant; WCL, whole-cell lysate; WGA, wheat germ agglutinin.

Previous data confirm that FcRγ lies downstream of CD36, but it is unclear whether these proteins form a multimeric signaling complex. Therefore, we examined the composition of a potential CD36 signalosome in human platelets. Immunoprecipitation of CD36 from human platelet lysates confirmed that this scavenger receptor associates with Fyn and Lyn under both basal and stimulated conditions (Figure 3A). Furthermore, FcRγ was associated with CD36 under basal condition and there was no significant increase in association with oxLDL treatment (*P* < .07), although there was a trend for increased association. In contrast, JNK, which is also activated in platelets by oxLDL,^25^ did not form part of the complex (Figure 3A). Reverse immunoprecipitation of FcRγ confirmed its association with CD36, which was not significantly altered by oxLDL treatment (Figure 3B).

To confirm the interaction of CD36 and FcRγ in whole platelets, we used PLA based on in situ fluorescence. Washed platelets, adhered to oxLDL (100 μg/mL), were dual immunostained with antibodies for CD36 and FcRγ, and wheat germ agglutinin was used as a control stain for membrane glycoproteins. PLA-positive foci were detected for CD36 and FcRγ (0.3 ± 0.1 arbitrary units [AU]) and for CD36 and Lyn (0.12 ± 0.1 AU). PLA signals were absent when either the CD36 or FcRγ antibodies were used alone or when the secondary PLA probes were used alone (Figure 3C). As a negative control, we used an antibody to PKA as a protein that does not localize with CD36, and, as expected, we found no signal using antibodies to PKA and CD36. Together these findings suggest that CD36 and FcRγ are in functional proximity in situ.

**Figure 4.**
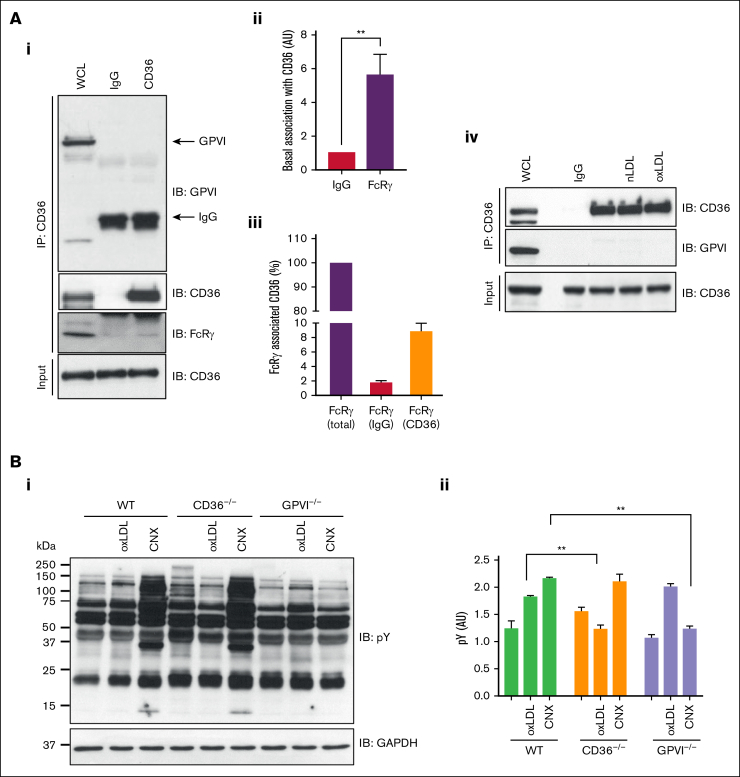
**CD36 association with FcRγ is independent of GPVI in platelets.** (A) CD36 was immunoprecipitated from washed human platelets (7 × 10^8^/mL) and immunoblotted for GPVI, CD36, and FcRγ. (i) Representative blots, (ii) densitometric analysis of FcRγ, and (iii) percentage of total FcRγ associated with immunoprecipitated CD36, from 5 independent experiments. (iv) As in subpanel i, except platelets were untreated or treated with oxLDL (50 μg/mL) or nLDL (50 μg/mL) for 15 seconds. Representative blots from at least 3 independent experiments. (B) Washed platelets (5 × 10^8^/mL) from WT, CD36^−/−^, and GPVI^−/−^ mice were either untreated or treated with oxLDL (50 μg/mL) or CNX (500 ng/mL), lysed, and immunoblotted for phospho-tyrosine. Membranes were stripped and reprobed with GAPDH antibody. (i) Representative blots and (ii) densitometry analysis for phospho-Syk from 3 independent experiments. ∗∗*P* < .01. CNX, convulxin; GAPDH, glyceraldehyde-3-phosphate dehydrogenase; IgG, immunoglobulin G; WCL, whole-cell lysate.

FcRγ is an established effector of GPVI signaling in platelets and therefore we explored the possibility that GPVI may contribute to our observations. Immunoprecipitation of CD36 from human platelets did not reveal an association with GPVI under basal conditions (Figure 4Ai) but confirmed association with FcRγ. Given that FcRγ can interact with multiple proteins in platelets, we used densitometry to show that only a small proportion of the adapter was in a complex with CD36 (Figure 4Aiii). Treatment with LDL did not change this interaction (Figure 4Aiv). To confirm that GPVI did not influence signaling, we used murine platelets deficient in CD36 or GPVI. These experiments revealed that oxLDL induced protein tyrosine phosphorylation in GPVI-deficient but not CD36-deficient platelets (Figure 4B). The platelet GPVI surface expression was unaffected in WT and CD36^−/−^ groups but was diminished in GPVI^−/−^ (supplemental Figure 2). Although these data indicated that CD36 did not require GPVI, it was possible that CD36 could influence GPVI dimerization, which is important for its signaling capacity.^26^ To address this, we used an Affimer, D18, which binds specifically to dimeric but not monomeric GPVI to see, by flow cytometry, whether oxLDL induced GPVI clustering.^27^ Activation of human platelets with collagen-related peptide XL (10 μg/mL) caused a significant increase in binding of dimer-specific Affimer, D18, but did not increase scaffold control or pan-GPVI Affimer M17 (supplemental Figure 3). In contrast to GPVI stimulation, oxLDL (50 μg/mL) did not significantly increase D18 binding. These data suggest that oxLDL-stimulated tyrosine phosphorylation of FcRγ is dependent on CD36 and does not require or activate GPVI.

Next, we used a Jurkat T-cell–line model system, which we have previously used to study FcRγ signaling, to investigate the capacity for oxLDL to signal through CD36 and FcRγ in the absence of GPVI.^28^ The assay uses a NFAT/activator protein 1 (AP-1) transcriptional reporter to produce a luciferase signal in response to combined Ca^2+^ and mitogen-activated protein kinase signaling, both of which are downstream of ITAM signaling. Jurkat T cells do not express CD36, GPVI, α_IIb_β_3_, or FcRγ, but do express the structurally and functionally homologous T-cell receptor ζ-chain that can substitute for FcRγ.^29^ Transfection of CD36 and NFAT/AP-1–luciferase constructs resulted in basal NFAT/AP-1 activity, which was increased significantly by stimulation with oxLDL (supplemental Figure 4A). Deficiency of the SFK family member Lck, the Syk family member ZAP-70, and SLP-76 ablated the NFAT/AP1 response, without affecting the expression of CD36 (supplemental Figure 4B). These model cell line data confirm that oxLDL can signal via CD36 through the canonical activation of SFK and ITAM signaling.

### FcRγ is required for oxLDL-CD36–induced tyrosine kinase signaling and platelet activation

**Figure 5.**
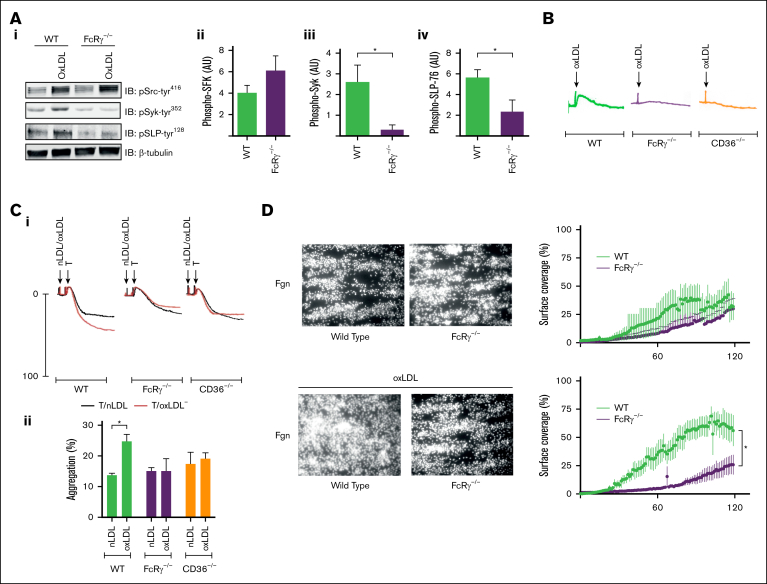
**OxLDL-induced platelet activation requires the FcRγ.** (A) Washed platelets (5 × 10^8^/mL) from WT and FcRγ^−/−^ mice were either unstimulated or stimulated with oxLDL (50 μg/mL) for 15 seconds and then lysed. Lysates were immunoblotted for pSrc-tyr^416^, pSyk-tyr^352^, pSLP-76-tyr^128^, and β-tubulin. Representative blots (i) and densitometric analysis (ii-iv) of 4 independent experiments. ∗*P* < .05. (B) Washed platelets (2.5 × 10^8^/mL) from WT, FcRγ^−/−^, and CD36^−/−^ mice were incubated with a combination of apyrase (2 U/mL), indomethacin (10 μM), and EGTA (1 mM) for 15 minutes and then stimulated with oxLDL (50 μg/mL) for 2 minutes. Representative traces of 4 independent experiments. (C) Washed platelets (2.5 × 10^8^/mL) from WT, FcRγ^−/−^, or CD36^−/−^ mice were either incubated with oxLDL (50 μg/mL; red line) or nLDL (50 μg/mL; black line) for 30 seconds followed by stimulation with thrombin (0.02 U/mL) and aggregation was recorded for 4 minutes. Representative aggregation traces of 3 independent experiments (i) and aggregation (percent) (ii) expressed mean ± standard error of the mean (SEM; n = 3). ∗*P* < .05. (D) Whole blood from WT and FcRγ^−/−^ mice was incubated with oxLDL (100 μg/mL) or vehicle for 1 minute and then perfused at arterial shear 1000 s^−1^ for 2 minutes over immobilized fibrinogen (100 μg/mL). Images of adherent platelets were taken by fluorescence microscopy. Representative images of arterial flow experiments (left panels) and surface coverage (percent) presented as a function of time (right panels). Data are expressed mean ± SEM (n = 5). ∗*P* < .05. Fgn, fibrinogen; pSLP-76-tyr^128^, phospho–SLP-76-tyr^128^; pSrc-tyr^416^, phospho–Src-tyr^416^; pSyk-tyr^352^, phospho–Syk-tyr^352^; T, thrombin.

To investigate the functional importance of the FcRγ to platelet hyperactivity induced by oxLDL, we used mice in which FcRγ had been genetically deleted. Immunoblotting confirmed the absence of FcRγ in these mice (supplemental Figure 5) but no change in the expression of CD36 (supplemental Table 1). Expression levels of important platelet receptors, including the loss of GPVI, were similar to those previously reported.^30^ We tested the effects of FcRγ deficiency on platelet signaling induced by oxLDL, reasoning that in the absence of FcRγ, the signaling response upstream of FcRγ would be maintained, whereas those downstream would be lost. Consistent with human platelets, we found that activatory tyrosine (tyrosine^416^) phosphorylation of SFK in response oxLDL in WT murine platelets was maintained in FcRγ^−/−^ platelets (Figure 5A). We then examined proteins known to be phosphorylated downstream of FcRγ. oxLDL induced the phosphorylation of Syk (phospho–Syk-tyr^352^) and its downstream effector SLP-76 (phospho–SLP-76-tyr^128^) in WT platelets but not in FcRγ^−/−^ platelets (Figure 5Ai-iv). These data support the findings that SFKs lie upstream of FcRγ phosphorylation by oxLDL, whereas Syk and SLP-76 are phosphorylated downstream of this ITAM-containing adapter.

Having found that tyrosine phosphorylation of key activator proteins was lost in the absence of FcRγ, we reasoned that the activatory effects of oxLDL would be compromised. We used a series of increasingly physiological in vitro models to examine this possibility. To confirm the functional response of FcRγ was initiated by CD36, we also investigated platelets from CD36^−/−^ mice. Consistent with previous observations in human platelets,^7^ oxLDL induced shape change in WT platelets, which was absent in FcRγ^−/−^ and CD36^−/−^ platelets (Figure 5B). Furthermore, oxLDL was unable to potentiate thrombin-induced aggregation in the absence of the CD36 receptor or FcRγ (Figure 5C). The aggregation of washed platelets in response to thrombin was comparable in WT and FcRγ^−/−^ mice. However, FcRγ^−/−^ platelets were unable to aggregate in response to GPVI stimulation by collagen (supplemental Figure 6). These data indicate that the signaling and functional activities of platelet CD36 in response to oxLDL require FcRγ.

Finally, we examined the importance of FcRγ in thrombus formation using an in vitro flow assay (Figure 5D). Under arterial shear (1000 s^−1^), the preincubation of WT whole blood with oxLDL (100 μg/mL) led to a significant increase in maximum surface area coverage (51.3% ± 6.9% to 68.9% ± 10%; *P* < .05). In contrast, oxLDL failed to potentiate thrombosis when tested in combination with blood from FcRγ^−/−^ mice (43.5% ± 3.7% to 36.9% ± 4.1%; *P* < .07). We found no overall differences in platelet deposition and thrombus formation in WT and FcRγ^−/−^ mice in the absence of oxLDL (Figure 5D). Thus, under experimental conditions, FcRγ is required for oxLDL-mediated platelet activation and aggregation.

### FcRγ deficiency protects against oxLDL-induced thrombosis

**Figure 6.**
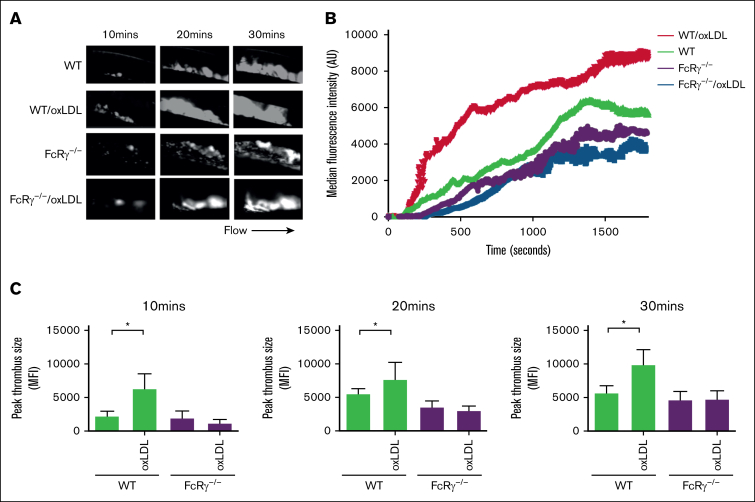
**FcRγ promotes thrombosis in response to oxLDL.** WT and FcRγ^−/−^ mice were injected with oxLDL (2.5 mg per kg body weight) or phosphate-buffered saline vehicle, followed by FeCl_3_ (5%) injury and in vivo thrombosis assessed by intravital microscopy. (A) Representative fluorescence images of thrombi formed under different conditions are shown over the course of 30 minutes after vascular injury. Black arrow shows the direction of blood flow. (B) Representative median integrated fluorescence signals of rhodamine G obtained from an individual carotid thrombus under different conditions. (C) Quantification of median integrated fluorescence signals of peak thrombus size at 10, 20, and 30 minutes after vascular injury taken from WT and FcRγ^−/−^ mice (n = 5) for each treatment. ∗*P* < .05.

Finally, to address the importance of FcRγ for oxLDL-induced in vivo thrombosis*,* we used intravital microscopy after the infusion of oxLDL into mice followed by ferric chloride–induced carotid artery injury. Tail-vein injections of oxLDL (2.5 mg/kg body weight)^31^ into WT mice accelerated after injury thrombotic responses at all time points compared with phosphate-buffered saline injection (negative control; Figure 6). Next, we repeated these experiments in FcRγ^−/−^ mice, reasoning that the absence of the signaling adapter would diminish the prothrombotic effects of oxLDL. Under control conditions (buffer infusion), the absence of FcRγ led to a modest but not significant reduction in thrombosis when compared with WT mice. However, deficiency in platelet FcRγ abolished the ability of oxLDL to enhance thrombosis at all time points after injury (*P* < .01).

## Discussion

A substantial body of research suggests that dyslipidemia-associated oxLDL promote thrombosis through modulating platelet function, including the augmentation of platelet activation, the inhibition of platelet inhibitory signaling, and the generation of procoagulant platelets.^32^^,^^33^ The scavenger receptor CD36, which belongs to the class B scavenger receptors, has emerged as a pivotal receptor linking these 3 prothrombotic functions. We have shown previously that CD36 signals through a process dependent on SFK and PLCγ2,^7^ although, the precise mechanism by which CD36 uses these tyrosine kinases to transmit extracellular lipid stress signals to activate platelets remains unclear. Studies in nucleated cells have shown that CD36 initiates signaling in a ligand-specific manner by forming cooperative partnerships with other receptors and adapter proteins.^17^ In platelets, proteins containing immunoreceptor ITAMs facilitate tyrosine kinase signaling downstream of several key receptors. Given these observations, we wished to uncover the mechanisms underlying CD36 signaling and how it affects the ability of oxLDL to promote thrombosis. Here, we provide multiple lines of evidence to suggest that the ITAM-containing protein FcRγ is key to oxLDL-induced platelet activation. We show that oxLDL promotes the tyrosine phosphorylation of FcRγ but not FcγRIIA, which is lost under conditions of CD36 deletion or inhibition. A small pool of CD36 forms a signaling complex with FcRγ, and the stimulation of CD36 by oxLDL results in the phosphorylation of the ITAM and the recruitment and phosphorylation of Syk. Critically, the genetic deletion of FcRγ prevents oxLDL-induced platelet activation, aggregation, and thrombosis both in vitro and in vivo.

Proteomic analysis of platelets treated with oxidized phospholipids commonly found in oxLDL revealed the tyrosine, threonine, and serine phosphorylation of a significant number of platelet proteins.^34^ The use of SFK kinase inhibitors indicated that Fyn and/or Lyn were functionally connected to a range of signaling proteins, including extracellular signal-regulated kinase, p38, Mkk4/JNK, Vav1/3, PLCγ2, Syk, and RhoA.^7^^,^^9^^,^^35^^,^^36^ The sequential activation of SFKs and Syk in response to oxLDL, downstream of CD36, is similar to GPVI and α_IIb_β_3_, with ITAM adapter proteins facilitating the transmission of receptor signaling into the interior of a cell. Given that ligation of CD36 leads to homoclustering and heteroclustering with other receptors,^37^ we hypothesized that ITAM coreceptor cooperation was necessary for CD36 signaling in platelets. The treatment of platelets with oxLDL reversibly phosphorylates FcRγ in a SFK-dependent manner, providing a functional explanation for early observation that SFKs were constitutively associated with platelet CD36.^38^ We confirmed that Lyn and Fyn are part of the constitutive multiprotein complex with CD36, which was populated with Syk upon oxLDL treatment. In situ PLA demonstrated the close proximity of CD36 to FcRγ in human platelets, confirming association in whole cells. Given the differences in copy number of these proteins, with human platelets expressing CD36 up to 15 000 copies and FcRγ 1500 copies,^39^ it suggests that only a small pool of CD36 is linked to FcRγ and that other receptors may also form partnerships with platelet CD36. For example, a CD36/TLR2/TLR6 complex is responsible for platelet activation by oxidized phospholipids.^40^ Our work further enhances this concept, by identifying a new partner, FcRγ, and excluding 2 others, FcγRIIA and GPVI. We observed oxLDL/CD36-mediated tyrosine phosphorylation in WT mice, which do not express FcγRIIA, indicating that CD36 can signal in the absence of that coreceptor. Moreover, we found no difference in oxLDL-induced signaling in response in murine platelets expressing FcγRIIA as a transgene compared with WT platelets, and found FcγRIIA was not phosphorylated within human platelets. Although GPVI is associated with FcRγ, we detected no association between CD36 and GPVI in human platelets, and critically, observed that oxLDL was able to induce whole-cell tyrosine phosphorylation in platelets deficient in GPVI. Furthermore, oxLDL induced ITAM signaling via CD36 in the Jurkat T-cell model cell line, which lacks GPVI expression. Thus, our data indicate that CD36 can signal through FcRγ in the absence GPVI. The crucial role of CD36 in FcRγ phosphorylation was confirmed, with the effects of oxLDL recapitulated by a CD36-specific ligand, oxPC_CD36_, and absent in platelets lacking CD36. Interestingly coimmunoprecipitation studies indicated that although oxLDL stimulated the phosphorylation of FcRγ, the level associated with CD36 was statistically unchanged, suggesting a constitutive interaction. However, we found this response to be variable, with oxLDL clearly increasing the association of CD36 and FcRγ in some donors. Given that the level of expression of CD36 influences platelet response to oxLDL, it is possible that the association is linked to levels of CD36 on the platelet surface.^39^

The pathophysiological importance of the CD36-FcRγ interaction was explored with murine platelets deficient in FcRγ. Interestingly, the absence of FcRγ did not affect CD36 expression levels, suggesting a different relationship between FcRγ and CD36 compared with GPVI, which requires FcRγ for its expression. The absence of the ITAM had no effect on oxLDL-induced SFK phosphorylation but resulted in the loss of Syk and SLP-76 phosphorylation, consistent with their roles upstream and downstream of the ITAM, respectively. Furthermore, oxLDL potentiated platelet aggregation and enhanced thrombosis under flow in whole blood, which was prevented by the deletion of either CD36 or FcRγ. To validate this conclusion, we examined in vivo thrombosis after infusing oxLDL into FcRγ-deficient mice. Given the intricate relationship between FcRγ and GPVI in driving thrombosis, which appears to be context dependent, we used a mild FeCl_3_ injury model. This model has been shown to induce similar levels of platelet accumulation and time to occlusion in FcRγ-deficient and WT mice.^41^ By infusing oxLDL, we replicated the prothrombotic phenotype observed in several hyperlipidemic models, which led to accelerated thrombosis. Crucially, platelet hyperactivity and the associated accelerated thrombosis induced by oxLDL were prevented in FcRγ-deficient mice. Therefore, the significant number of responses that have been reported to be linked to CD36-tyrosine kinase signaling in platelets, including shape change, spreading, reactive oxygen species generation and procoagulant activity,^7^^,^^42^^,^^43^ could suggest a role for ITAM involvement in these processes and ultimately in arterial thrombosis linked to oxidative lipid stress.

oxLDL are found within atherosclerotic plaques, in the circulation of patients with acute coronary syndrome,^5^ with their concentrations positively correlating with thrombus severity in ST-elevated myocardial infarction.^44^ This suggests that platelets could be exposed to oxLDL both acutely after plaque rupture but also chronically in the circulation. The nature of the responses to these different scenarios is still unclear. We have shown previously that longer term exposure of platelets to oxidative lipid stress reduces platelet sensitivity to the inhibitory action of nitric oxide and prostacyclin.^31^^,^^45^ The current data suggest that acute exposure of platelets to oxLDL allows a CD36-FcRγ complex to promote platelet activation in a tyrosine kinase–dependent manner. Indeed, tyrosine kinase inhibitors can abolish oxLDL-induced platelet adhesion, spreading, shape change, α-granule secretion, and phosphatidylserine exposure,^7^^,^^9^^,^^46^^,^^47^ suggesting that this signaling mechanism is critical to CD36-mediated platelet activation. Interestingly, the involvement of SFK and Syk in CD36 signaling is not only limited to platelet responses, with these kinases playing critical roles in fatty acid uptake in adipocytes,^48^ endothelial cell migration in angiogenesis,^49^ reactive oxygen species generation in vascular smooth muscle cells,^50^ and macrophage foam cell formation.^51^

Taken together, our data support the existence of a multiprotein CD36 signalosome that comprises the receptor, SFKs (Fyn and Lyn), and the adapter FcRγ. oxLDL binding to CD36 results in FcRγ phosphorylation and facilitates Syk recruitment and phoshporylation, which likely underlies previously reported downstream signaling to PLCγ2.^7^^,^^52^ Therefore, identification of this signalosome could offer opportunities for controlling lipid-induced thrombosis. Direct inhibition of CD36 is not a feasible strategy given its wide expression and roles in metabolism. However, a more nuanced strategy for selective targeting of CD36 activities could be to disrupt specific components of distinct CD36 signalosomes to prevent unwanted platelet activation.

Conflict-of-interest disclosure: The authors declare no competing financial interests.

The current affiliation for M.S.H. is Centre for Biomedical Science Research, School of Health, Leeds Beckett University, Leeds, United Kingdom.

## Supplementary Material

Supplemental Methods, References, Tables, and Figures
