# Supplementary material for: The immune receptor FcRγ-chain mediates CD36-induced platelet activation and thrombosis by oxidized low-density lipoproteins
Source: Blood Adv. 2025 Jul 18;9(22):5774–86. doi: 10.1182/bloodadvances.2024015652 (PMC12661298; doi:10.1182/bloodadvances.2024015652)

METHODS

Platelet preparation

Human washed platelets (WP) were isolated from blood taken from drug-free volunteers, as previously described[1]. All human work was approved by the Hull York Medical School Ethics Committee. Human blood was taken by clean venepuncture using acid citrate dextrose (ACD; 29.9mM sodium citrate, 113.8mM glucose, 72.6mM sodium chloride and 2.9mM citric acid, pH 6.4) as anticoagulant. Platelet-rich plasma (PRP) was obtained by centrifugation of whole blood at 200g at room temperature for 20 minutes. PRP was treated with citric acid (0.3M) and centrifuged at 800g for 12 minutes. The platelet pellet was then suspended in wash buffer (36mM citric acid, 10mM EDTA, 5mM glucose, 5mM KCl, 9mM NaCl) and spun once more at 800g for 12 minutes. Platelets were finally re-suspended at the indicated concentrations in modified Tyrodes buffer (150mM NaCl, 5mM HEPES, 0.55mM NaH_2PO_4, 7mM $NaHCO_3$, 2.7mM KCl, 0.5mM $MgCl_2$, 5.6mM glucose, pH7.4).

Murine blood obtained by cardiac puncture was taken into PPACK under terminal CO_2 narcosis[2]. PRP was obtained by centrifugation of whole blood at 300*g* for 10min at room temperature. The PRP was then treated with citric acid (0.3M) and centrifuged at 1000*g* for 6 minutes at room temperature. The pellet was resuspended in wash buffer, spun again at 1000g for 6 minutes and then finally resuspended at the indicated concentration in modified Tyrodes buffer.

For signalling and shape change experiments, human or murine platelets were incubated with ethylene-glycol-bis-tetraacetic acid (EGTA; 1mM), indomethacin (10µM) and apyrase (2U/mL) for 15 minutes before experimentation.

Platelet aggregation

Washed murine platelets (2.5×10^8 platelets/mL) were either stimulated with oxLDL (50µg/mL) in the presence of a combination of EGTA (1mM), apyrase (2U/ml) and indomethacin (10µM) to induce a shape change response, or incubated with oxLDL/nLDL (50µg/mL) at 37°C for 30 seconds, followed by stimulation with thrombin (0.02U/ml). Aggregation or shape change were monitored under constant stirring for 4 minutes or 2 minutes, respectively, using a Chronolog Dual Channel Platelet Aggregometer.

Platelet flow assays

Vena8 biochips (Cellix; Dublin, Ireland) were coated with fibrinogen (100µg/mL) for 12 hours and blocked with BSA (10mg/mL) for 1 hour. Murine whole blood was incubated with $DiOC_6$ (1µM) and treated with oxLDL (100µg/mL) for 1 minute. Flow was performed for 2 minutes at $1000s^{-1}$. Images of stably adhered platelets and thrombi were captured using fluorescence microscopy and analysed using ImageJ software. Data are presented as surface area coverage (%), as the software could not fully discriminate between single platelets and platelet aggregates[2].

Immunoprecipitation and Western blotting

For signaling studies, washed platelets ($5x10^8$/mL, unless stated otherwise) were pretreated with EGTA (1mM), , apyrase (2U/mL) and indomethacin (10µM) to prevent secondary mediated signalling. Platelets were treated with nLDL or oxLDL (0-100µg/mL; for 15-300 seconds) or with PAPC or $oxPC_{CD36}$ (0-50µM; for 15-300 seconds) before termination with either Laemmli buffer (4% SDS (w/v), 10% 2-mercaptoethanol (v/v), 20% glycerol (v/v), 50mM Tris base, trace bromophenol blue, pH 6.8) for whole cell lysate studies or with ice cold lysis buffer (150mM NaCl, 10mM Tris base, 1mM EGTA, 1mM EDTA, 1% Igepal (v/v), 1mM PMSF, 2.5mM Na_3VO_4, 0.5% protease inhibitor cocktail (v/v), 0.5% phosphatase inhibitor cocktail (v/v), pH 7.4) for immunoprecipitation studies. In some cases, platelets were pre-incubated with PP2 (20µM), PP3 (20µM) or Dasatinib (10µM) for 3 minutes or FA6.152 (1µg/ml), SSO (50µM) for 5 minutes before addition of oxLDL or $oxPC_{CD36}$. Platelets were lysed with an equal volume of ice-cold IP lysis buffer and left on ice or rolled in the cold room for 30 minutes to allow for complete lysis. The lysates were then cleared by centrifugation at 12,000g at 4°C for 10 minutes to remove platelet debris. The lysates were incubated on ice for 30 minutes and then pre-cleared using Protein A-Sepharose beads (or Protein G-Sepharose beads for CD36 (N15) antibody experiments) for 1 hour at 4°C. Proteins were then immunoprecipitated overnight at 4°C using Protein A-Sepharose beads (or Protein G-Sepharose beads for CD36 (N15) antibody) in combination with anti-FcRγ-chain

antibody (1µg), anti-Syk antibody (4D10, 1µg) for human studies, anti-Syk antibody (5F5, 2µg) for murine studies, anti-CD36 antibody (FA6.152, 2µg) for human studies or anti-CD36 antibody (N15, 2µg) for murine studies, anti-FcγRIIA antibody (2µg). Proteins were separated by sodium dodecylsulfate polyacrylamide gel electrophoresis (SDS-PAGE) and transferred to low fluorescent polyvinylidene difluoride (PVDF) membranes. Membranes were then dried for 1 hour followed by re-activation with methanol, blocked with 5% milk for 45 minutes and then incubated with anti-phospho-tyrosine antibody (4G10, 1:1000), anti-FcRγ-chain antibody (1:1000), anti-Syk antibody (4D10, 1:1000) for human studies, anti-Syk antibody (5F5, 1:1000) for murine studies, anti-Fyn antibody (1:1000), anti-Lyn antibody (1:1000), anti-CD36 antibody (H300, 1:1000) for human studies, anti-CD36 antibody (N15, 1:500) for murine studies, anti-FcγRIIA antibody (1:500), anti-JNK antibody (1:1000), anti-GPVI antibody (1:750), anti-GAPDH antibody (1:1000), anti-phospho-Src^{Tyr416} antibody (1:1000), anti-phospho-Syk^{Tyr352} antibody (1:1000), anti-phospho-SLP-76^{Tyr128} antibody (1:1000) or anti-β-tubulin antibody (1:1000) overnight at 4°C. Immunoblots were then incubated with either HRP-conjugated or fluorescently labelled secondary antibodies, followed by ECL detection or imaging using the LI-COR Odyssey CLx system, according to the manufacturer's instructions.

Proximity ligation assay

Protein interactions in whole, intact platelets were visualised by *in situ* fluorescence. Washed human platelets (3 x 10^6 per coverslip) were adhered to oxLDL (100µg/ml) coated coverslips for 60 minutes at 37°C. Samples were then incubated overnight at 4°C with one of the following antibody combinations: anti-CD36 antibody (FA6.152, 1:500) plus anti-FcRγ-chain (1:100), anti-CD36 (FA6.152, 1:500) plus anti-Lyn antibody (1:200), anti-CD36 (FA6.152, 1:500) plus anti-PKA RII antibody (1:100), anti-CD36 (FA6.152, 1:500) alone, anti-FcRγ-chain (1:100) alone or antibody diluent containing no antibodies. Samples were processed using the Duolink In Situ kit following the manufacturer's instructions. Images were captured using the Zeiss LSM 710 confocal microscope under 63x magnification[3].

Flow cytometric analysis

For platelet surface receptors, whole blood from wild-type, $FcR\gamma^{-/-}$ and $Fc\gamma RIIA^{+/+}$ animals was stained with 1µg/mL of fluorophore-conjugated antibodies against the indicated platelet surface receptors for 10 minutes at RT, then analysed by flow cytometry. In separate experiments, whole blood from wild-type, $CD36^{-/-}$ and $GPVI^{-/-}$ mice was stained with 1µg/mL of fluorophore-conjugated anti-GPVI and analysed by flow cytometry.

Intravital imaging of *in vivo* thrombosis

The left carotid artery of anaesthetised mice was exposed, and mice were injected with Rhodamine G, followed by either oxLDL (2.5mg/kg body weight) or an equal volume of PBS through the tail vein. 10 minutes after the injection of oxLDL, injury was induced by applying 1x2 mm filter paper saturated with anhydrous $FeCl_3$ (10%). The filter paper was placed on the right adventitial surface of the vessel for 1 minute and then removed. Thrombosis was recorded using high-speed intravital microscopy for up to 40 minutes. The integrated intensity value (Median Fluorescence Intensity) of the thrombus was measured over time and the time taken to reach peak was determined. Four individual mice were used for each condition.

NFAT/AP-1 luciferase reporter assay

The Jurkat T cell line was cultured and transfected as previously described[4]. The NFAT/AP-1luciferase assay and the β-galactosidase assay, used to normalize for transfection efficiency, were carried out as previously reported[4]. The human CD36 expression construct in the pEF vector was kindly provided by Dr Fedor Berditchevski (University of Birmingham).

Statistical analysis

Experimental data was analysed by Graphpad Prism 6 (La Jolla, CA, USA). Data are presented as mean ± standard error of the mean (SEM) of at least three different experiments (unless otherwise stated). Differences between groups were calculated using Mann-Whitney U Test or

Kruskal-Wallis Test for non-parametric testing and statistical significance accepted at $P \leq 0.05$. All studies were approved by the Hull York Medical School Ethics and University of Leeds Research Ethics committees.

References

1. Wraith, K. S. *et al.* Oxidized low-density lipoproteins induce rapid platelet activation and shape change through tyrosine kinase and Rho kinase–signaling pathways. *Blood* **122**, 580–589 (2013).
2. Magwenzi, S. *et al.* Oxidized LDL activates blood platelets through CD36/NOX2-mediated inhibition of the cGMP/protein kinase G signaling cascade. *Blood* **125**, 2693–2703 (2015).
3. Raslan, Z., Magwenzi, S., Aburima, A., Taskén, K. & Naseem, K. Targeting of type I protein kinase A to lipid rafts is required for platelet inhibition by the 3′,5′-cyclic adenosine monophosphate-signaling pathway. *J. Thromb. Haemost.* **13**, 1721–1734 (2015).
4. Tomlinson, M. G. *et al.* Collagen promotes sustained glycoprotein VI signaling in platelets and cell lines. *J. Thromb. Haemost.* **5**, 2274–83 (2007).

SUPPLEMENTARY DATA

Supplementary Table 1

**Receptor**	**Wild type (MFI)**	**FcRγ chain$^{-/-}$ (MFI)**	**P- value**
GPIb	173.5±15.4	177.3±13.9	0.86
Integrin αIIb	91.3±2.8	90.5±3.5	0.87
Integrin β3	269.5±42.4	191±36.9	0.17
CD36	675±211	713.7±284	0.96
GPVI	97.4± 8.8	1.1±1.7	0.002

Supplementary Table 2

**Receptor**	**Wild type (MFI)**	**FcγRIIA chain$^{+/+}$ (MFI)**	**P- value**
GPIb	190.3±10.1	175.7±8.7	0.40
Integrin αIIb	107.4±2.33	105.3±1.5	0.41
Integrin β3	329±1.9	364±28.8	0.18
CD36	625±190	613±213	0.46
GPVI	96.3±3	100.7±3.7	0.4

Supplementary Tables. Expression of major platelet receptors on murine platelets
PRP from either wild-type, FcRγ-chain$^{-/-}$ (Table 1) or FcγRIIA$^{+/+}$ (Table 2) incubated with the appropriate antibodies and expression analysed by flow cytometry. Data are expressed as mean±SEM MFU of 5 independent experiments.

Supplementary Figure 1: Immunoblotting of platelet lysates for CD36
Washed human platelets, WT murine platelets and $CD36^{-/-}$ platelets ($5x10^8$/ml) were lysed with Laemmli buffer, separated by SDS-PAGE and immunoblotted with anti-CD36. Representative blot of 3 independent experiments

Supplementary Figure 2: Flow cytometric analysis of murine platelet GPVI
Murine blood was taken into tri-sodium citrate (109mM) as anticoagulant. For platelet surface receptors, whole blood was stained with 1µg/ml of anti-GPVI antibody. Data to the indicated platelet surface receptors for 10 minutes at RT and then analysed by flow cytometry. Data is presented as mean fluorescence intensity (MFI) form three individual mice of each strain.

Supplementary Figure 3: Flow cytometric analysis of platelet GPVI dimerization
Washed human platelets ($1x10^6$/ml) were treated with CRP-XL (10µg/ml), oxLDL (50µg/ml) or nLDL (50µg/ml) for 20 minutes in the presence or either scaffold, M17 (pan GPVI monomer/dimer) or D18 (GPVI dimer specific) Affimers conjugated with Alexa Fluor 488 (10µg/ml) and fluorescent signal was assessed by flow cytometry.

Supplementary Figure 4: CD36 and oxLDL induce signaling in the Jurkat T-cell line model
(A) The Jurkat T-cell line deficient in Lck, Zap40 or SLP76 was transfected with a Ca^{2+}/mitogen-activated protein kinase-responsive NFAT/AP-1-luciferase reporter construct, a β-galactosidase construct, a CD36 expression construct, or empty vector control. Cells were either left unstimulated or stimulated with 50µg/mL oxLDL, lysed, and assayed for luciferase and β-galactosidase. Results in all panels are expressed as mean ± standard deviation (SD) (n=4). *$P<0.05$. **(B)** as in (A) except surface expression of CD36 was examined by flow cytometry (n=4).

Supplementary Figure 5: Immunoblotting of murine platelet lysates for $FcR\gamma^{-/-}$.
Murine platelets from WT and $FcR\gamma^{-/-}$ mice ($5x10^8$/ml) were lysed with Laemmli buffer, separated by SDS-PAGE and immunoblotted with anti- FcRγ. Representative blot of 3 independent experiments

Supplementary Figure 6: Platelet aggregation in WT and $FcR\gamma^{-/-}$ murine platelets.
Murine wild type and $FcR\gamma^{-/-}$ platelets ($2.5x10^8$/ml) were stimulated with thrombin or collagen and aggregation was then measured under constant stirring (1000 rpm) at 37°C for 4 minutes. Representative aggregation traces of four independent experiments**.**

PLA Images

(i) CD36-FcRγ

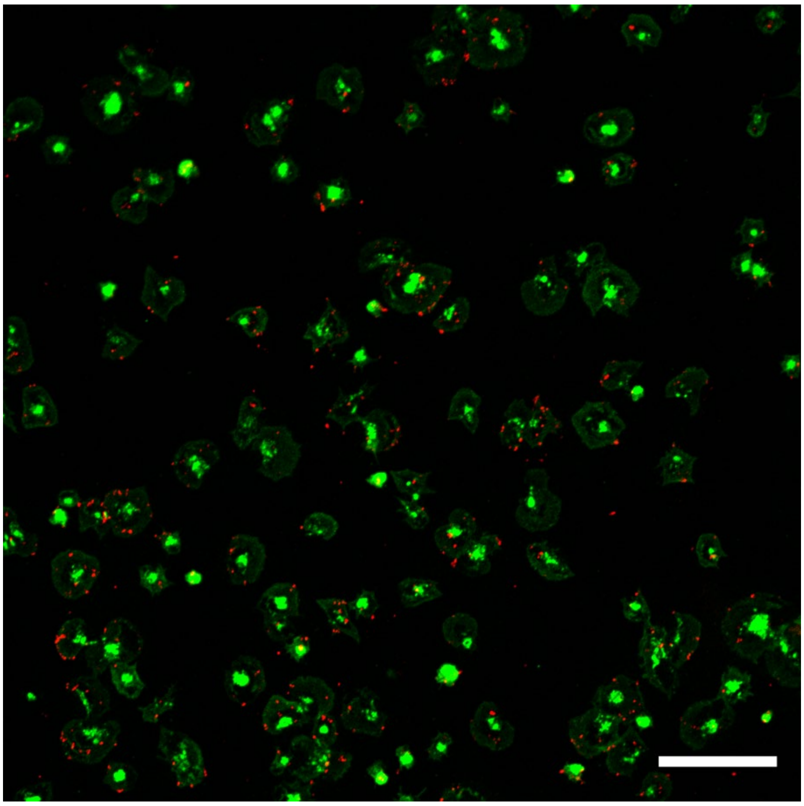

(ii) CD36-Lyn

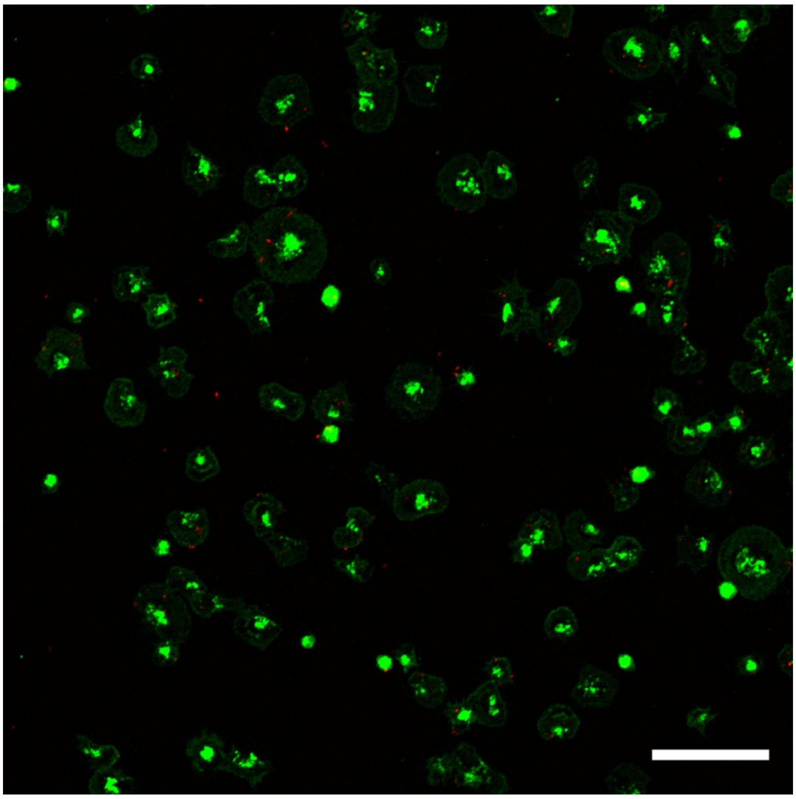

(iii) CD36 alone

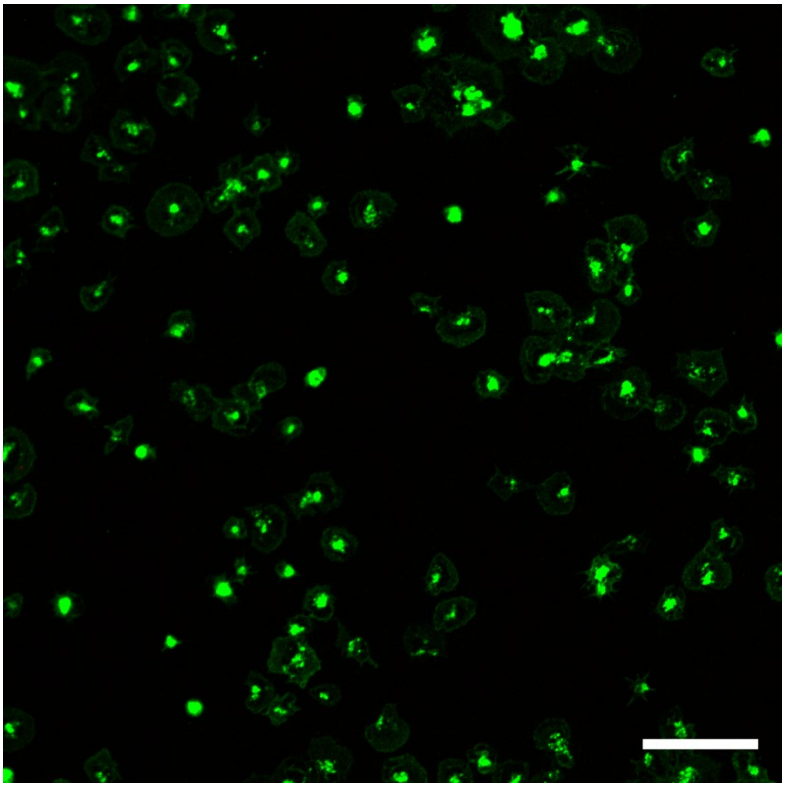

(iv) CD36-PKA

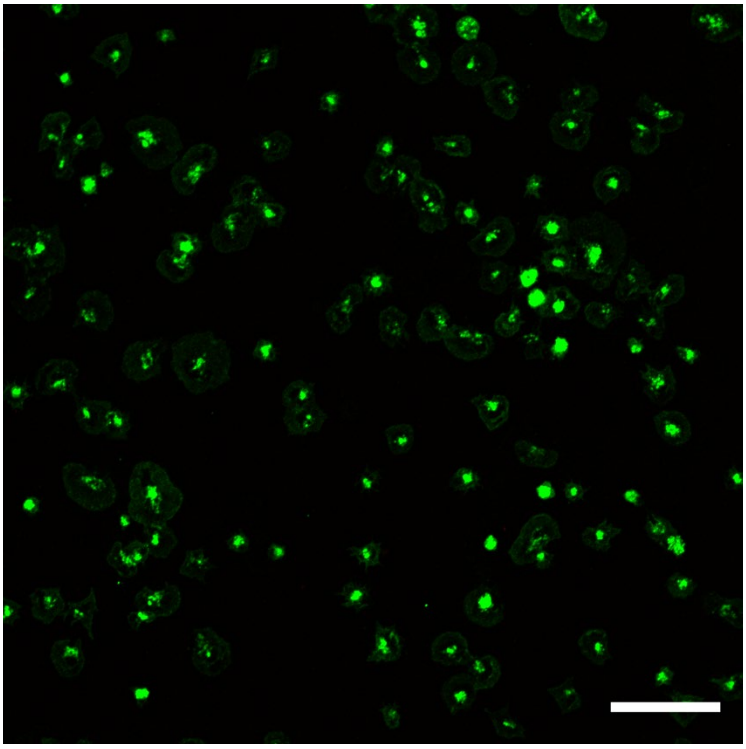

(v) FcRγ alone

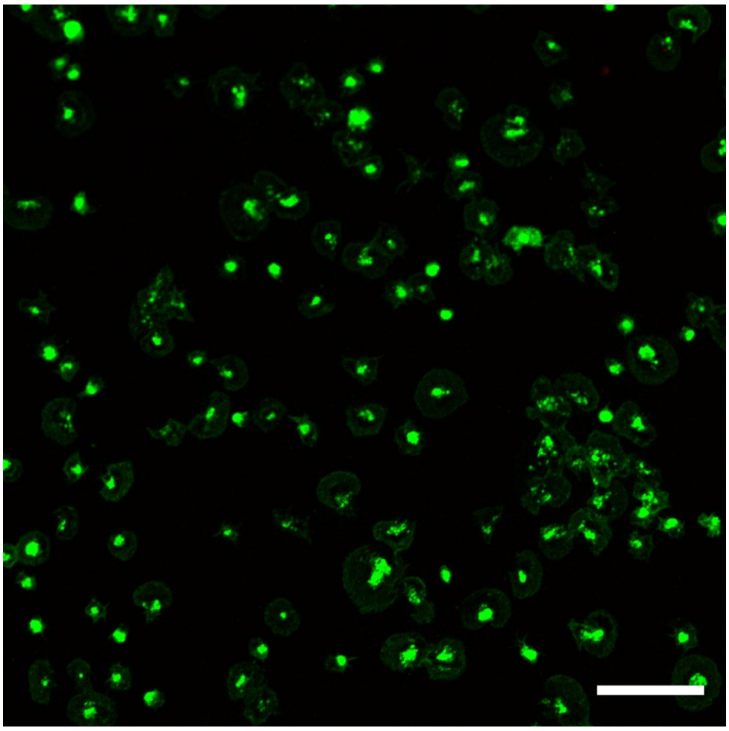

(vi) Secondary probes

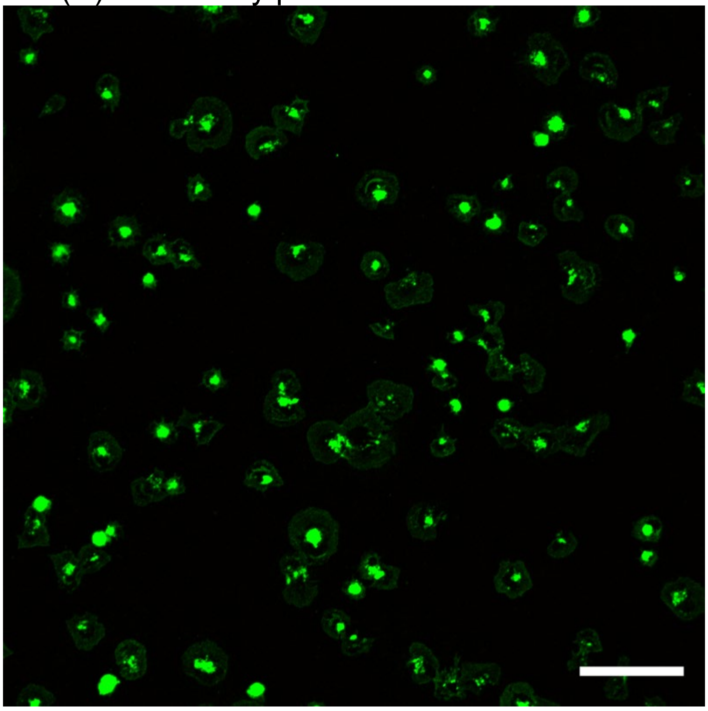

Supplement: Supplemental Methods, References, Tables, and Figures [file BLOODA_ADV-2024-015652-mmc1.pdf]
